# Metabolomics acts as a powerful tool for comprehensively evaluating vaccines approved under emergency: a CoronaVac retrospective study

**DOI:** 10.3389/fimmu.2023.1168308

**Published:** 2023-07-14

**Authors:** Xinyu Liu, Congshu Xiao, Pengwei Guan, Qianqian Chen, Lei You, Hongwei Kong, Wangshu Qin, Peng Dou, Qi Li, Yanju Li, Ying Jiao, Zhiwei Zhong, Jun Yang, Xiaolin Wang, Qingqing Wang, Jinhui Zhao, Zhiliang Xu, Hong Zhang, Rongkuan Li, Peng Gao, Guowang Xu

**Affiliations:** ^1^ CAS Key Laboratory of Separation Science for Analytical Chemistry, Dalian Institute of Chemical Physics, Chinese Academy of Sciences, Dalian, China; ^2^ Liaoning Province Key Laboratory of Metabolomics, Dalian, China; ^3^ Department of Infection, The Second Hospital of Dalian Medical University, Dalian, China; ^4^ University of Chinese Academy of Sciences, Beijing, China; ^5^ Hangzhou Health-Bank Medical Laboratory Co., Ltd., Hangzhou, China; ^6^ Clinical laboratory, Affiliated Dalian Hospital of Shengjing Hospital of Chinese Medical University, Dalian, China; ^7^ Nursing Department, Anshan Infectious Disease Hospital, Anshan, China; ^8^ Internal Department, Women and Children’s Hospital of Anshan City, Anshan, China; ^9^ Clinical laboratory, The Second Hospital of Dalian Medical University, Dalian, China; ^10^ Shanghai Institute for Biomedical and Pharmaceutical Technologies, Shanghai, China

**Keywords:** COVID-19, CoronaVac, vaccine, metabolomics, lipidomics, immune response

## Abstract

**Introduction:**

To control the COVID-19 pandemic, great efforts have been made to realize herd immunity by vaccination since 2020. Unfortunately, most of the vaccines against COVID-19 were approved in emergency without a full-cycle and comprehensive evaluation process as recommended to the previous vaccines. Metabolome has a close tie with the phenotype and can sensitively reflect the responses to stimuli, rendering metabolomic analysis have the potential to appraise and monitor vaccine effects authentically.

**Methods:**

In this study, a retrospective study was carried out for 330 Chinese volunteers receiving recommended two-dose CoronaVac, a vaccine approved in emergency in 2020. Venous blood was sampled before and after vaccination at 5 separate time points for all the recipients. Routine clinical laboratory analysis, metabolomic and lipidomic analysis data were collected.

**Results and discussion:**

It was found that the serum antibody-positive rate of this population was around 81.82%. Most of the laboratory parameters were slightly perturbated within the relevant reference intervals after vaccination. The metabolomic and lipidomic analyses showed that the metabolic shift after inoculation was mainly in the glycolysis, tricarboxylic acid cycle, amino acid metabolism, urea cycle, as well as microbe-related metabolism (bile acid metabolism, tryptophan metabolism and phenylalanine metabolism). Time-course metabolome changes were found in parallel with the progress of immunity establishment and peripheral immune cell counting fluctuation, proving metabolomics analysis was an applicable solution to evaluate immune effects complementary to traditional antibody detection. Taurocholic acid, lysophosphatidylcholine 16:0 sn-1, glutamic acid, and phenylalanine were defined as valuable metabolite markers to indicate the establishment of immunity after vaccination. Integrated with the traditional laboratory analysis, this study provided a feasible metabolomics-based solution to relatively comprehensively evaluate vaccines approved under emergency.

## Introduction

A new coronavirus, the severe acute respiratory syndrome coronavirus 2 (SARS-CoV-2), was identified at the end of 2019, and the resulting coronavirus disease (COVID-19) pandemic has spread over 3 years (1). This pandemic still sporadically emerges due to the frequently emerging variants and the unavailability of efficient antiviral drugs (2, 3). Vaccination was thought to be the most powerful tool for arresting the plague when the first outbreak of COVID-19 worldwide (4). In the past 3 years, over 200 candidate vaccines had been developed. Some of them had been approved for emergency use in specific areas or by meeting specified criteria during that time.

The development and approval of human use vaccines usually need several years (5). Although the commercial vaccines were approved under stringent regulations, some of them also needed further improvement with years of efforts considering immunological effects (6). The sudden pandemic had shortened the laboratory-to-market cycle of SARS-CoV-2 vaccines. The same situation was also the case for the corresponding SARS-CoV-2 antibody detection kits. What made the situation worse was that there was no commercial coronavirus vaccine on the market before the COVID-19 pandemic. In other words, human beings had no experience in coronavirus vaccine development and appraisals before 2020 (5).

CoronaVac (Sinovac Life Sciences, Beijing, China) was first approved by China and contained inactivated SARS-CoV-2 isolated at the early stage of the domestic plague (7). The primarily recommended vaccination program was a two-dose strategy with an, at least, 28-day interval (8). This vaccine was primarily applied to healthy young people. Due to various uncertainties, CoronaVac was not recommended to old people and persons with chronic diseases at its primary application stage. This plight has made the relevant population be lagging behind the national vaccination project. To date, CoronaVac is demonstrated to be safe for recipients with different biological and pathological backgrounds and is immunologically acceptable (9–15). Unfortunately, drawing this conclusion costs over 3 years with endeavors of over 400 clinical trials and real-world studies. This situation poses a great challenge to seek a solution to help scientists to evaluate vaccine effects confidently and timely when we face another new infectious pandemic.

Metabolomics, focusing on changes of small molecules in a given system, is an omics that directly mirrors the phenotypes (16). It provides a glimpse of the entire biological adaptive process after a specific stimulus from the facet of the metabolome dynamics (17, 18). Metabolomics strategy has been employed to evaluate immune responses of some vaccines, and helped to explore immune effects that could not be uncovered by antibody detection or immune cell function appraisals (19–23). Not limited to that, metabolomics can help to find early biomarkers to indicate drug toxicities sensitively. The U.S. Food and Drug Administration (FDA) and European Medicines Agency had approved several metabolite biomarkers for regulatory use in apprising drug nephrotoxicity (24).

**Figure 1:**
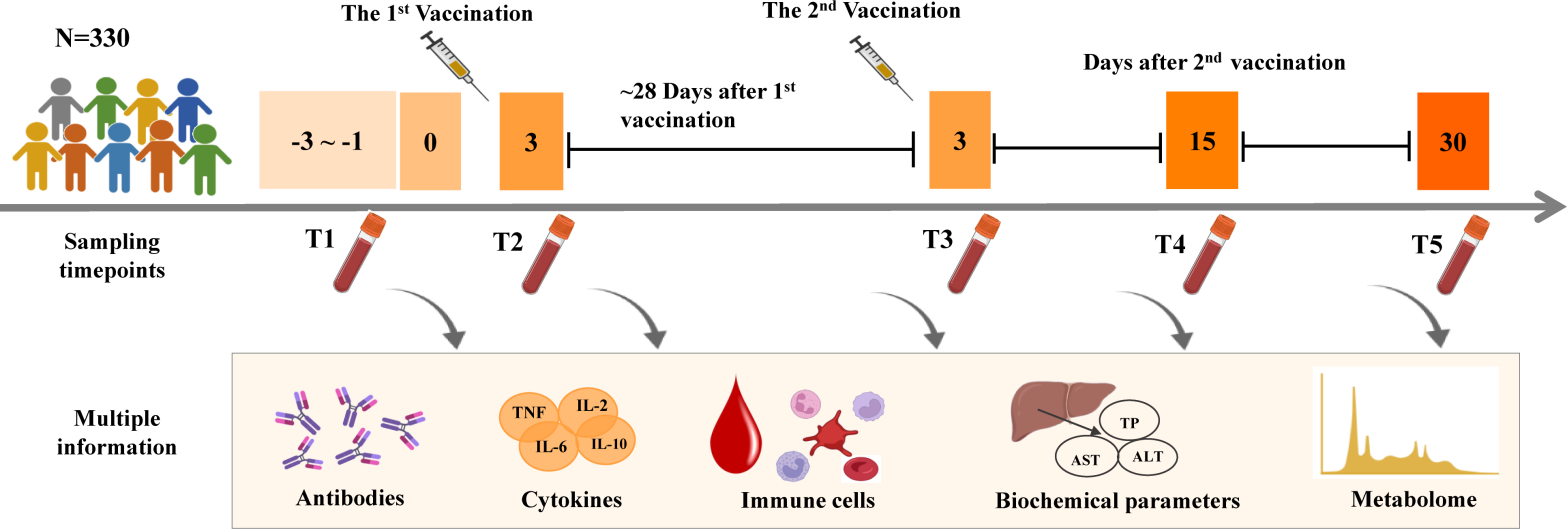
Experimental design and sampling points of serum and plasma samples in the study.

It costs almost 3 years of massive studies to prove CoronaVac is safe and immunologically acceptable. This time-consuming process is not compatible with the intention of emergent approval of vaccines. It is very valuable to find clues to prove CoronaVac’s usefulness at its early application stage. The relevant tactic will be in favor to direct future emergent vaccine and drug approval. To this end, we retrospectively studied the sera from 330 Chinese volunteers collected at 5 different time points before and after CoronaVac vaccination in 2020 (**Figure 1**). Routine clinical laboratory tests and mass spectrometry-based metabolomics and lipidomics analyses were performed for each sample. The metabolic data after inoculation were compared against that of the pre-inoculation to evaluate any potential unexpected effects in light of metabolic phenotype shift. Furthermore, the samples were randomly divided into independent discovery and validation sets to investigate and confirm vaccination-associated dynamic metabolic responses. The key metabolic modules and pathways related to immune effects were explored. Finally, metabolite markers indicating immunity establishment were defined and validated. Our data indicated that this metabolomics study not only opens up a new way to monitor the vaccination-associated metabolic perturbations but also provides metabolite markers for immune effect evaluation complementary to traditional antibody detection methods.

## Methods

### Studied subjects

The study was approved by The Ethics Committee of the 2^nd^ Affiliated Hospital of Dalian Medical University (2021-No.032). 148 male and 182 female Chinese volunteers from Dalian and Anshan were enrolled in this study. All the subjects were randomly divided into the discovery set (n=164) and the validation set (n=166) with matched genders and ages. Written informed consent was provided by each participant. Each participant received the recommended two doses of CoronaVac (8). Fasting blood specimens were sampled using vacuum tubes with EDTA-Na_2_ as the anticoagulant (for plasma) or tubes containing separation gel (for serum) (Sanli Medical Technology, Hunan, China). Blood samples were collected at five different time points throughout the study, namely before the first vaccination (T1), 3 days after the first vaccination (T2), as well as 3 days (T3), 15 days (T4), and 30 days (T5) after the second vaccination (**Figure 1**).

### Routine clinical laboratory analysis

Blood cell analysis was performed by employing a UniCe® DxH 800 hematology analyzer (Beckman Coulter, Brea, CA) with reagents from the identical manufacturer. Blood glucose, total protein, albumin, total bilirubin, cholesterol, triglyceride, total bilirubin, alanine aminotransferase, aspartate aminotransferase, urea, and creatinine were analyzed by an ADVIA 2400 biochemical analyzer (Siemens Healthineers, Erlangen, Germany). The reagents were purchased from Zhongyuan Biotechnology (Chongqing, China). Serum SARS-CoV-2 Immunoglobulin G (IgG) and Immunoglobulin M (IgM) were detected by an SF100 immunofluorescent analyzer (B&C Biological Technology, Shanghai, China) using the kits provided by the same manufacturer. The concentrations of interleukin 2 (IL-2), interleukin 4 (IL-4), interleukin 6 (IL- 6), interleukin 10 (IL-10), tumor necrosis factor-alpha (TNF-α), and interferon-gamma (IFN-γ) were quantitatively determined using a CBA kit (Human Th1/Th2 Cytokine Kit, JiangXi Cellgene, NanChang, China) analyzed by a BD FASCantoII flow cytometry (San Jose, California).

### Untargeted metabolomics and lipidomics analysis

LC-MS based untargeted serum metabolomics analysis was carried out following our previous report (25). Briefly, 400 μL of methanol/acetonitrile (1:1, v/v) was added to 100 μL of serum. After vortexed for 60 seconds, the mixture was centrifuged. Each supernatant was dried in a vacuum centrifuge and then reconstituted in 50 μL of acetonitrile/water (2:8, v/v). For metabolomics analysis, each 3 μL reconstructed sample was loaded on a BEH C8 column (for ESI^+^ analysis) and an HSS T3 column (for ESI^-^ analysis) (2.1 mm × 50 mm, 1.7 µm particle size) (Waters Corp, Milford, USA) to be separated respectively. The elution was directed to a Q Exactive mass spectrometry (Thermo Fisher Scientific, Rockford, USA) for detection. The flow rate was set at 0.4 mL/min, and the column temperature was 60 °C. Mobile phase A for positive and negative modes was water containing 0.1% (v/v) formic acid and 6.5 mM ammonium bicarbonate respectively. Mobile phase B for positive and negative modes was acetonitrile containing 0.1% (v/v) formic acid and 95% (v/v) methanol containing 6.5 mM ammonium bicarbonate respectively. The positive gradient program started at 5% B maintained for 0.5 min, linearly increased to 60% B at 2 min, then linearly increased to 100% B in 6 min, held for 2 min, then dropped to 5% B in 0.1 min, and held for 2 min. The negative gradient program started at 2% B, maintained for 0.5 min, linearly increased to 40% B at 2 min, then linearly increased to 100% B in 6 min, held for 2 min, then dropped sharply to 2% B in 0.1 min, and held for 2 min. The total run time of positive or negative mode was 12.0 min.

The LC-MS based untargeted serum lipidomics analysis was carried out as described previously (26). Briefly, 240 μL of methanol was added into 20 μL of serum. After brief vortex, 800 μL of methyl tert-butyl ether (MTBE) was added to the mixture. After centrifugation, 350 µL aliquot of each upper layer solution was separated by a BEH C8 (2.1 × 100 mm, 1.7 µm particle size) (Waters Corp, Milford, USA) column. The eluted components were analyzed by a Q Exactive mass spectrometry (Thermo Fisher Scientific, Rockford, USA). The flow rate was 0.3 mL/min, and the column temperature was 60 °C. Mobile phases A and B were acetonitrile/H20 (60:40, v/v) containing 10 mM ammonium acetate and IPA/CAN (90:10, v/v) containing 10 mM ammonium acetate respectively. The gradient program started at 50% B maintained for 1.5 min, linearly increased to 85% B at 9 min, then sharply increased to 100% B in 0.1 min, held for 1.9 min, then dropped sharply to 50% B in 0.1 min, and held for 1.9 min. The total run time of positive or negative mode was 13.0 min.

To monitor the robustness of the metabolomics and the lipidomics analysis, pooled quality control (QC) samples were constructed and inserted into the analysis queue every ten runs. Peak detection and integration were performed by using Tracefinder software (Thermo Fisher Scientific, Waltham). For comparison purposes, each peak area was normalized to the sum area of peaks in the corresponding sample. The metabolite identification was based on our in-house database (27). The lipid identification was first performed by LipidSearch software (Thermo Fisher Scientific, Waltham), and then cross-checked against an open database utilizing specific retention time, exact m/z and MS^2^ fragments.

### Statistical analysis

For univariate analysis, the Wilcoxon Signed-rank test for paired samples was conducted using R software version 4.1.1. The Benjamini-Hochberg (BH) method was utilized to control the false discovery rate (FDR). Adjusted *p-*values below 0.05 were considered significant. Spearman correlation analysis was used to investigate the relationship between two specific parameters when needed. Partial least square discriminant analysis (PLS-DA) was performed by using SIMCA-P v14.1 (Umetrics, Sweden). Differential correlation analysis was performed using the R package of DGCA (28). Metabolite pairs with differential correlation (*p* < 0.05) were subjected to multiscale embedded correlation network analysis (using R package of MEGENA) (29).

## Results

### Immunological and hematological responses to CoronaVac vaccination

**Figure 2:**
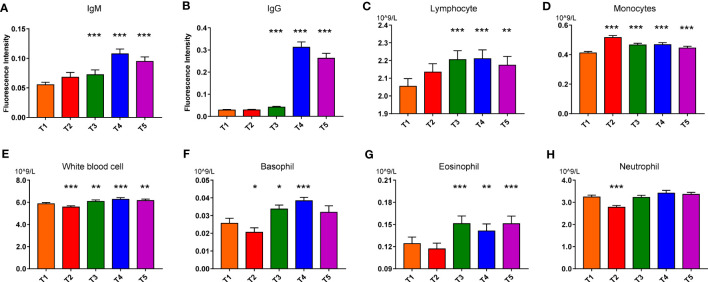
IgM **(A)**, IgG **(B)**, Lymphocyte **(C)**, Monocytes **(D)**, White blood cell **(E)**, Basophil **(F)**, Eosinophil **(G)**, Neutrophil **(H)** variations in response to vaccination in the discovery set. Paired nonparametric test, *: *p*FDR<0.05, **: *p*FDR<0.01, and ***: *p*FDR<0.001, compared with T1 (T1 to T5: before the first vaccination (T1), 3 days after the first vaccination (T2), 3 days (T3), 15 days (T4), and 30 days (T5) after the second vaccination; IgG: Immunoglobulin G; IgM: Immunoglobulin M).

The 330 Chinese volunteers were randomly divided into the discovery set (n=164) and the validation set (n=166) with matched genders and ages. Serum immunoglobulin G (IgG) and immunoglobulin M (IgM) were monitored at five different time points individually (**Figure 1**). The detectable IgM was produced as early as 3 days after the first dose vaccination in some recipients (**Figure 2A**). While the IgG antibodies could be mainly detected after the second dose vaccination (**Figure 2B**). For T5, the serum antibody positive rate of the whole vaccinated population was around 81.82% (83.54% and 80.12% in the discovery and validation sets, respectively, **Table S1**), which was comparable to the corresponding reports (30, 31). In this light, the following study involved in immunity establishment was based on the data at T5 with at least one positive serotype of antibody.

Then routine hematological and biochemical results of the discovery set were shown in **Table S2**. Nearly all blood cell counting and liver function parameters slightly fluctuated after vaccination. Serum total protein, glucose and cholesterol decreased, while triglyceride increased a little bit after vaccination (**Figures S1A–H**). Counting of the white blood cells (WBCs), neutrophil, basophil and eosinophil decreased conspicuously after 3 days of the first dose vaccination and then increased after the boost dose. While the monocyte number significantly elevated after 3 days of the first vaccination and then gradually declined, presenting an opposite change trend compared to the other immune cells (**Figures 2C–G**). Of note, all the routine test parameters were within their reference intervals, coinciding with the ~3 years observation in that the CoronaVac did not bring about apparent pathological hematological and biochemical damage. Similar results were also found in the validation set (data not shown). Moreover, no difference was found in the plasma cytokine concentrations (TNF-α, IFN-γ, IL-2, IL-4, IL- 6, and IL-10 (**Figures S1I–N**) before and after vaccination. This meant that the inactivated virus was not capable of stimulating extra cytokine secretion as the active virus did (32).

### Vaccination-associated metabolic responses

**Figure 3:**
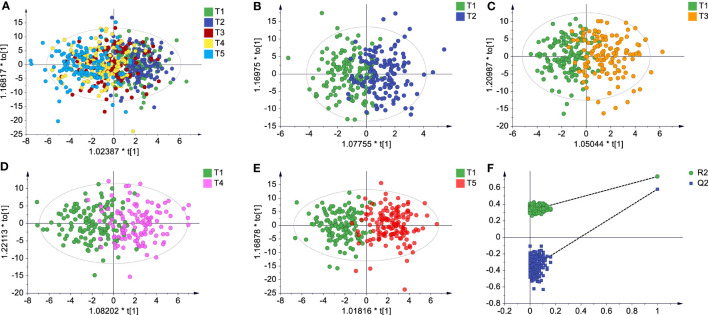
Global metabolic difference of subjects with different metabolic variations in response to vaccination in discovery set. **(A)** Score plot of OPLS-DA model for all subjects at different sampling times (T1~T5). **(B–D)** Score plot of OPLS-DA model for T1 vs. T2, T1 vs. T3, and T1 vs. T4. **(E, F)** Score plot and cross validation of OPLS-DA model for T1 vs. T5. In **(A–E)** UV scaling was used, no overfitting was found.

To gain a holistic view of the systemic responses to the CoronaVac, the serum metabolic and lipidomic profiling data were acquired based on our reliable metabolomics and lipidomics strategies (**Figure S2**). The 246 identified metabolites of the discovery set were subjected to principal component analysis (PCA). The 5 time points data of all the recipients before and after the vaccination overlapped each other (**Figure 3A**), implying no evident adverse effect occurred after the vaccination (33). The separation trend gradually became obvious when the data of T2 to T5 were compared individually with that of T1 after supervised multivariate analysis (**Figures 3B–E**). The conspicuous metabolic shifts were found at 15 and 30 days after the second dose vaccination (**Figures 3D–F**), suggesting the metabolic adaptation to the vaccine was still active after one month of the boost dose. Similar metabolic changes were also observed in the validation set (**Figure S3**). For the sole lipidomics data, no obvious separation trend could be discerned even between T1 and T5 (data not shown), which hinted lipid responses to the vaccination were less sensitive. In the subsequent analysis, the two omics data were combined and called “metabolic profiling (MP) data”.

In the early stage application of CoronaVac, there was a safety concern about old people (34). But, the ~3-year observation data demonstrated that this concern was not necessary even for old people with various chronic diseases (9–15). To address this topic, we compared the MP data between old people and young people. No matter what time point data were considered, no separation could be discerned in the corresponding PCA plots (**Figure S4**). Even the MP data of young or old people were compared in the light of before and after inoculation, there was no separation in the relevant PCA score plots (**Figure S5**). These results indicated that all the recipients responded similarly to the vaccine irrespective of their age distribution. Thus, we could conclude that the CoronaVac did not elicit unexpected side effects, at least, in view of perturbations on the metabolome.

According to the ~3 years’ observation data, the immunity was well established after 30 days of the boost dose (12, 30, 34). In this line, the associations between the key metabolites (with *p*FDR<0.05 in the discovery set) and immune state were further explored based on the MP data of T5. Among the differential metabolites extracted from the comparison between T5 and T1 in the discovery set, 213 metabolites (*p*FDR<0.05) could be confirmed to be perturbated significantly in the validation set. Correlation analysis exhibited that most of the perturbed metabolites fluctuated in parallel with the counting of WBCs with varied degrees (**Figure S6**). The correlation between polar metabolites, *e.g.*, amino acids, energy-metabolism-related metabolites, microbe-related metabolites as well as bile acids, and blood immune cells as well as antibodies was mainly positive. Specifically, taurocholic acid (TCA) and taurodeoxycholic acid (TDCA) showed the strongest correlation with serum IgG and IgM. While the negative correlation was found between most of the fatty acids and blood immune cells as well as antibodies, except for polyunsaturated eicosanoic acids and docosanoic acids. For lipids, lysophosphatidylcholines (LPCs) and lysophosphatidylethanolamines (LPEs) presented remarkably positive linkages with serum IgG and IgM. While sphingomyelin showed negative correlations with blood immune cells. Interestingly, TGs of more than 4 double bonds were negatively related to antibody levels. Whereas TGs of less than 4 double bonds presented positive relationships with blood immune cells. Collectively, the CoronaVac triggered a complex adaptation process at the metabolite levels and the metabolite fluctuations were closely related to the blood immune cell numbers and antibody levels.

### Key metabolic modules and pathways related to the immune effect

**Figure 4:**
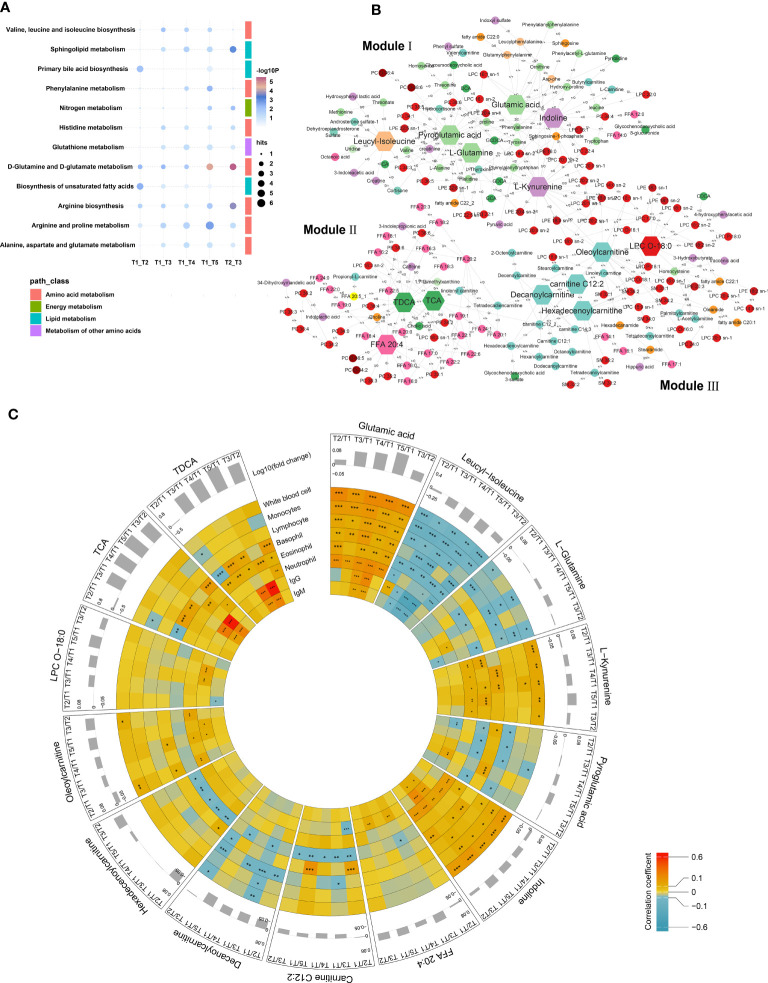
Key metabolic modules related to vaccine stimuli and crucial metabolites contributed to immune response to vaccination. **(A)** Pathway enrichment based on validated differential metabolites in the discovery and validation sets between T2, T3, T4 or T5 and T1, as well as between T2 and T3. **(B)** Differential metabolite correlation analyses of serum metabolites in T5 vs. T1 based on metabolomics profiling in the whole vaccinated population. Changes of correlation between metabolite-pairs in 30 days after the second vaccination (T5) relative to before vaccination (T1) were calculated. Those with differential correlations with *p* < 0.05 were subjected to construct co-expression network by employing Multiscale Embedded Gene Co-expression Network Analysis (MEGENA). The hub nodes were labeled with hexagon shape. **(C)** Associations between Hub metabolites and blood immune cells as well as antibody in different timepoints in the vaccinated population with at least a positive antibody at T5 (T2 vs. T1, T3 vs. T1, T4 vs. T1, T5 vs. T1 and T3 vs. T2) (Spearman correlation coefficient, *: p<0·05, **: p<0·01, ***: p<0·001).

To further confirm the progressively temporal changes of metabolic responses after vaccination, the pathway enrichment analysis was performed based on the validated differential metabolites and lipids at different time points. Rapid responses first occurred in the primary bile acid biosynthesis, unsaturated fatty acid biosynthesis and amino acid metabolic pathways 3 days after the first dose vaccination (T2). The perturbated processes after the boost dose (T3, T4 and T5) included sphingolipid metabolism, phenylalanine metabolism, arginine biosynthesis, arginine and proline metabolism, as well as alanine, aspartate and glutamate metabolism (**Figure 4A**), indicating the first dose and the second dose triggered different metabolic responses, which were more likely involved in immune memory settlement and activation.

To further comprehensively and reliably visualize the complex metabolic adaptation network (MAN) after vaccination and define the key metabolic modules related to immune effects, multiscale embedded gene co-expression network analysis was performed based on the metabolomics profiling of all subjects with at least one serotype positive antibody at T5 (**Figure 4B**). Three key modules were extracted. Glutamic acid, glutamine, pyroglutamic acid, indoline, kynurenine and leucyl-isoleucine were the hub nodes of Module I, and concurrently linked to dipeptides, bile acids, amino acids, LPCs, LPEs, PCs, as well as microbe-related metabolites. Taurine conjugated bile acids (TCA and TDCA) and FFA 20:4 as the hub nodes of Module II, and were mainly related to the metabolism of PCs, PEs, LPCs and FFAs, etc. The hub nodes of Module III included LPC O-18:0 and acylcarnitines (hexadecenoylcarnitine, oleoylcarnitine, decanoylcarnitine and carnitine C12:2) pathways and were mainly linked to LPCs, LPEs, SMs and long-chain acylcarnitines, etc. Notably, the alterations of major hub metabolites were significantly related to the counting of blood immune cells and the generation of antibodies (**Figures 4C**; **S6**). Lysophospholipids, especially LPCs and LPEs with carbon numbers of 16, 18, 20 and 22 entangled with all the key modules. Such results hinted lysophospholipids’ important roles in bridging metabolic adaptation and immunity establishment. The three modules shared similarities in that their hub metabolites were intricately connected by acylcarnitines, amino acids, lipids, bile acids, and so on. Collectively, a complex metabolic adaptation occurred at the global metabolic network level.

The hub metabolites were related to the immune cells in varying degrees (**Figure 4C**). Conspicuous associations were found between glutamic acid, glutamine, kynurenine, indoline, leucyl-isoleucine, pyroglutamic acid, acylcarnitines, as well as bile acids (TCA and TDCA) and the counting of immune cells in the whole observation period. Interestingly, glutamic acid, indoline, TCA and TDCA maintained a strong correlation with immune cells at 15 days after the boost dose, and presented a stronger correlation with IgG and IgM. The correlation was still evident at 30 days after the second dose. While unconjugated primary bile acid CDCA was found to be associated with white blood cell, monocyte and lymphocyte, which was different with conjugated primary bile acid TCA and TDCA, indicating different immune responses. These results suggested the hub metabolites were related to the reinforcement of immune effects.

When the perturbated metabolites were projected to concrete metabolic pathways, it was found that the metabolic disturbance mainly focused on the processes of glycolysis, tricarboxylic acid cycle, amino acid metabolism, urea cycle, and phospholipids metabolism (**Figure S7**). Pyruvic acid and lactic acid were significantly elevated after 3 days of the second dose vaccination, implying activated glycolysis. Amino acid metabolism was also significantly up-regulated. While the tricarboxylic acid cycle was down-regulated after vaccination. Decreased ratio of acetylcarnitine (Car C2:0) and propionylcarnitine (Car C3:0) to carnitine implied a reduced β-oxidation rate after vaccination (**Figure S7**) (35). The ratio of (Car C16:0+Car C18:1) to Car C2:0 was generally considered an indicator of carnitine palmitoyl transferase 2 (CPT2) activity associated with mitochondrial long-chain fatty acid oxidation (36). The reversely increased (Car C16:0+Car C18:1)/Car C2:0 demonstrated the decreased activity of CPT2, which also provided evidence for the metabolic shift from energy supply to building blocks supply. The sum of several lipid species presented increased serum levels after vaccination. The free fatty acids mainly showed a downregulated trend, except for FFA 16:1 and FFA20:4. For TGs, molecules with more than 4 double bonds were significantly decreased, but those with less than 4 double bonds increased after vaccination. The changes of SM, PC and PE were also found, but no obvious consistent change pattern could be discerned (**Figure S8**).

Nearly all the detectable taurine/glycine-conjugated primary/second bile acids were increased after vaccination, including TCA, GCA, CDCA and etc. Changes of the ratios among different bile acids indicated up-regulated bile acids metabolism, which reflected the active response of co-metabolism between the liver and the intestinal microbiota (**Figure S9**). Moreover, gut microbes-relevant tryptophan and phenylalanine metabolism memorably increased after vaccination.

It has been demonstrated that two-dose CoronaVac vaccination elicited proper immune memory (37). The metabolic difference between T1 and T5 represented the activation of vaccination-related immune memory. The metabolic difference between T2 and T3 was involved in primary antibody generation and the reinforcement of immune memory building. In this light, immune memory-related metabolites were explored. 60 of the verified differential metabolites overlapped between T2 vs. T3 and T1 vs. T5. These metabolites were mainly related to amino acid metabolism, such as arginine and proline metabolism, arginine biosynthesis, as well as glutamate metabolism (**Figure 4A**). It was further found that most of the 102 differential metabolites between T2 and T3 were perfectly covered by the MAN (**Figures 4B, C**). The module hubs, such as arginine, 4-hydroxyquinoline, glutamic acid, glutamine, indoline, taurocholic acid (TCA), taurodeoxycholic acid (TDCA) were perfectly covered.

### Immune-related metabolite marker discovery and validation

**Figure 5:**
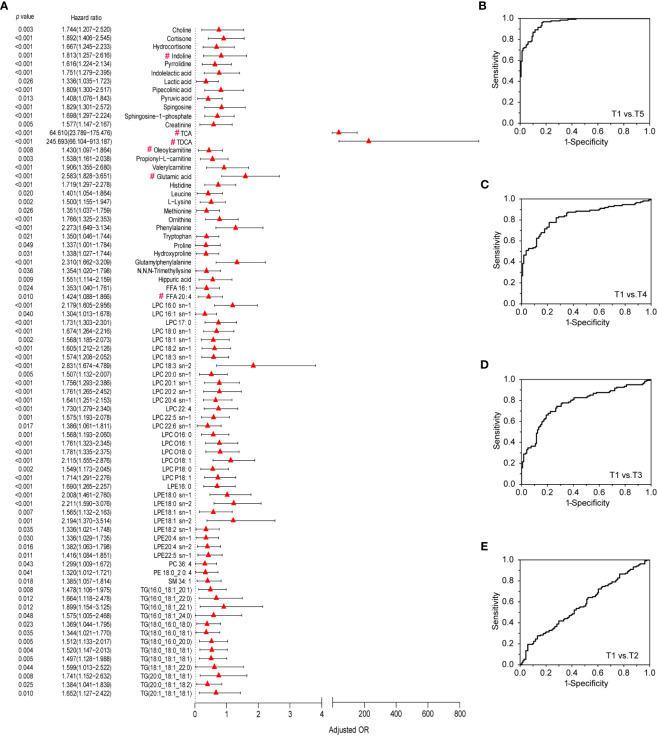
Differential metabolites that contribute to metabolic immune response to vaccination and ROC curves of the metabolic markers for the evaluation of immune response. **(A)** Validated differential metabolites in discovery and validation sets that contribute to metabolic immune response to vaccination based on data of the discovery set. Odds ratios (ORs) per 1 standard deviation increase in levels of differential metabolites between T1and T5 groups and 95% confidence interval (CI), which were adjusted by gender, age, BMI, metabolites marked with “#” were the Hub metabolites from **Figure 4B**. **(B–E)** ROC curves of metabolic markers for the evaluation of immune response between T5, T4, T3, or T2 and T1 in the discovery set.

To refine and focus on the crucial metabolites which contributed to immunity establishment, odds ratios (OR) and their 95% confidence intervals of the differential metabolites were calculated against genders, ages and BMIs. A total of 78 metabolites with OR values >1 were positively associated with the immune responses to the CoronaVac (**Figure 5A**). Most of these metabolites were the core metabolites in the MAN (**Figure 4B**). The 78 crucial metabolites in **Figure 5A** were subjected to binary logistic regression analysis. TCA, LPC 16:0 sn-1, glutamic acid, and phenylalanine were selected as efficient immune markers in the discovery phase. Among them, glutamic acid and TCA were the hubs of Module I and Module II, respectively. LPC 16:0 sn-1 was the linkage metabolites between Module I and Module II. The combination of the 4 metabolite markers could be used to distinguish T5 from T1 with high AUC (AUC=0.96), satisfied sensitivity (89.2%) and specificity (87.7%) (**Figure 5B**). When these metabolites were used to discriminate T4 from T1, the AUC was 0.833. The sensitivity and specificity were 80.3% and 73.2%, respectively (**Figure 5C**). The discrimination ability decreased when it came to T3 versus T1, as well as T2 versus T1 (**Figures 5D, E**, **Table S3**). Clearly, the discrimination abilities of the 4 metabolites exhibited time-course dependency with respect to immunity establishment. In the validation set, we further found that this combinational pattern of the 4 metabolite markers performed well to indicate immunity establishment (**Figures S10A–D**).

## Discussion

The outbreak of COVID-19 has brought about great social and economic changes to human beings. Vaccination is considered to be an effective way to control the spread and prevalence of COVID-19. A specific antibody is a traditional indicator of vaccine effects. Many methods have been developed for SARS-CoV-2 antibody detection (38) although the sensitivities and specificities of these methods varied greatly (39). It should be emphasized that simple antibody detection was not robust enough to evaluate vaccination efficiency because most of the detection kits were also approved for emergent use purposes. The FDA warned the public and healthcare providers that results from currently authorized SARS-CoV-2 antibody tests should not be used to evaluate a person’s level of immunity (https://www.fda.gov/medical-devices/safety-communications/antibody-testing-not-currently-recommended-assess-immunity-after-covid-19-vaccination-fda-safety).

The protection manners of vaccines were diverse. Evidence showed vaccination could not only trigger specific immunity by antibody generation but also elicit trained immunity or innate immune memory driven by epigenetic regulations and metabolic reprogramming (40, 41). The trained immunity was mediated by innate immune cells such as macrophages, monocytes, and natural killer (NK) cells. Such immune responses elicited non-specific memory and could combat secondary homologous or heterologous infections as exemplified by the influenza vaccine and BCG vaccine (42, 43). These protective effects could not be explained by specific antibodies but were achieved by innate immune components such as monocytes, macrophages, NK cells and proinflammatory cytokines (44). We found that cytokines were not significantly changed after vaccination, which was probably because the vaccine was made of inactivated virus. It was also reported that COVID-19 patients with agammaglobulinemia could recover without medical interventions, indicating that T cells alone could clear SARS-CoV-2 sufficiently (45, 46).

Immune cell counts fluctuated slightly but significantly after vaccination, suggesting immune response occurred after inactivated virus entry. Granulocytes decreased after the first dose, while lymphocytes, monocytes, and granulocytes memorably increased after the boost dose (**Figures 2C–H**). CoronaVac vaccination responses could be observed from the serum biochemical parameter changes (**Figure S1**, **Table S2**). The baseline lymphocyte counting was found to be significantly higher in COVID-19 survivors than in non-survivors. Decreased lymphocyte counting in the survivors could be improved during hospitalization, whereas severe lymphopenia was observed at the end stage of the non-survivors (47). These facts demonstrated the important roles of lymphocyte activities in the recovery of COVID-19 patients and the immune responses after the CoronaVac vaccination.

Advanced metabolomics approaches enabled a new possibility to define sequential immune responses at the metabolic level. Conspicuous metabolic changes were found after vaccination in this study. Energy metabolism-related pathways, amino acid metabolism, as well as microbial-related metabolism markedly shifted after vaccination. Glycolysis was critical for immune cell function and could be rapidly activated. The activated immune cells usually switched energy production from tricarboxylic acid cycle to glycolysis although the latter was not the most effective manner for ATP generation (48). Glycolysis provided considerable capacity for biosynthetic intermediates which enable immune cells to function properly (48). Coincidently, the reduction of metabolites in tricarboxylic acid cycle was also found in COVID-19 patients. It was ascribed to viral replication consuming malic acid and aspartate for purine and pyrimidine nucleotide biosynthesis (49). Fatty acid oxidation played an important role in functional immune cell generation and immune memory maintenance. Downregulated fatty acid oxidation had been found in activated effector T cells. This was understandable since the mobilization of immunity-related cells and antibody generation needed more bioenergy and biomaterials (40, 48). Similarly, the metabolism of amino acids (glutamine and arginine, etc.) was also found to play a role in sustaining proper immune function (50, 51), and closely related to immune responses. Many amino acids could be utilized to build antibodies and cytokines (52). Hence, such metabolic responses probably mirrored the immune cells’ activities during the establishment of immunity.

Bile acids, which are synthesized in the liver and further metabolized by intestinal microbiota, exert an extensive array of regulatory functions as signal molecules. Bile acid receptors (TGR5 and FXR) distribute in multiple organs, including the liver, intestine, adipose tissues as well as immune cells (53). It is reported that secondary bile acids are the most effective agonists of TGR5, and taurine and glycine-conjugated bile acids further augment the effects (53). Most of the detectable taurine/glycine-conjugated primary/second bile acids, such as TCA, TDCA and CDCA, were increased after vaccination in this study. Receptors for bile acids were expressed in several cells related to innate immunity (such as monocytes and macrophages etc.), and participated in the fine-tuning of these cells’ reactivity in response to endogenous antigens and bacteria (54). Thus, elevated bile acid metabolism probably contributed to immune cell activation, the establishment of immunity, as well as trained immunity or innate immune memory driven by metabolic reprograming.

**Figure 6:**
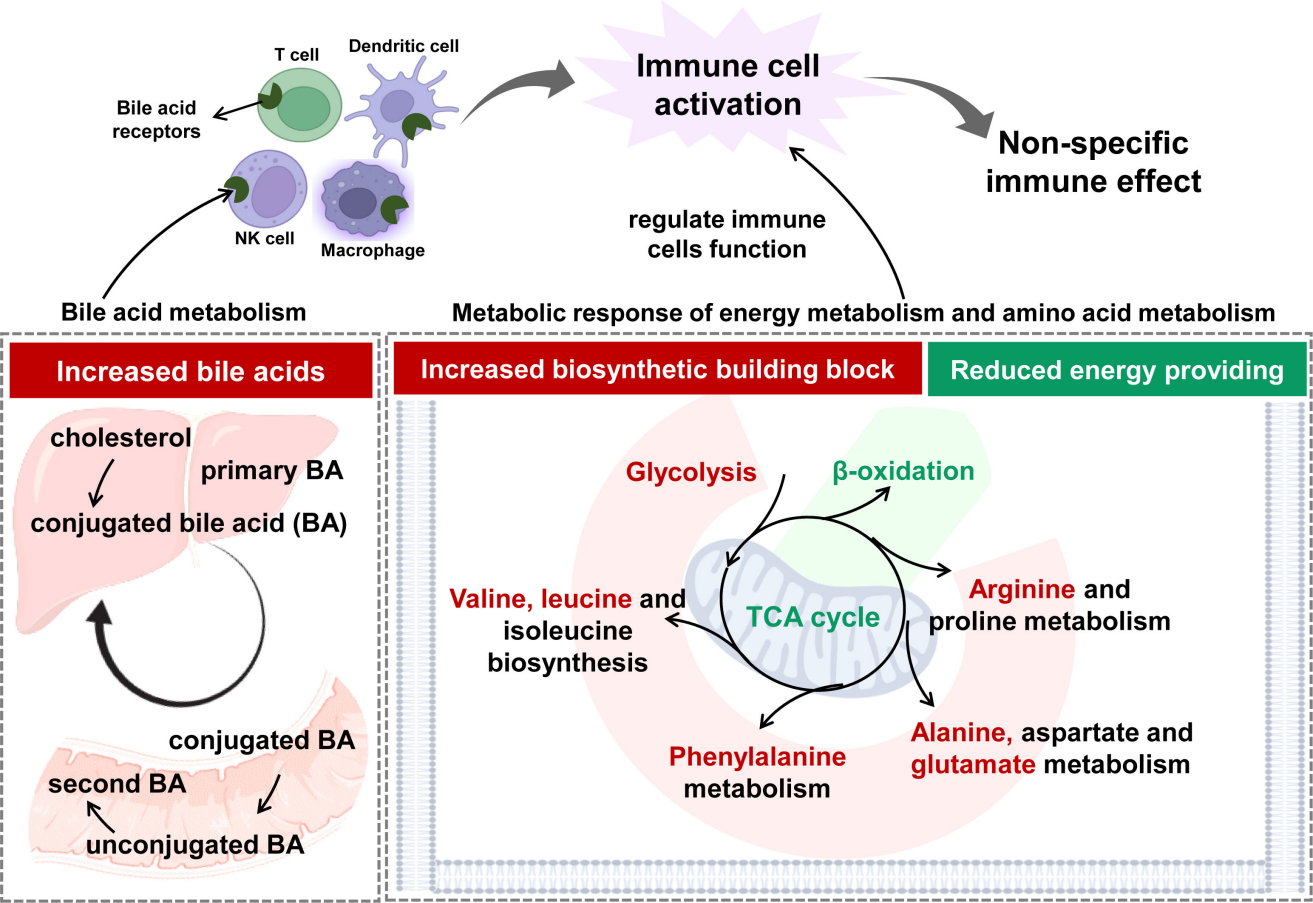
The speculated working model on the establishment of immune effect based on the response at metabolic level.

In this study, metabolomics analysis suggested that adaptive metabolism changes occurred in humans after vaccination. These alterations reflected the activated metabolic responses of immune cells. The speculated working model was summarized and presented in **Figure 6**. The pattern was different from those happening in COVID-19 patients in that CoronaVac did not elicit inflammatory responses (49). In summary, systemic metabolic response after a two-dose vaccination with inactivated SARS-CoV-2 in a Chinese population was demonstrated. A complex and conspicuous metabolic adaptation shift occurred in energy metabolism-related pathways (including glycolysis, tricarboxylic acid cycle, fatty acid oxidation), amino acid metabolism, as well as gut microbe-related metabolism. This dynamic adaptation was closely related to the counting of immune cells and probably contributed to immune memory and adaptive immunity. Furthermore, in the light of metabolomics, the biosafety of CoronaVac could be deduced based on metabolome change at the early stage. This study shed light on the interaction between inactivated SARS-CoV-2 and the human immune system at the metabolic level. Metabolomics analysis could benefit immune effect assessment by covering the whole stage of vaccination in a time-saving and result-convincing manner.

## Data availability statement

The data supporting the conclusions of this article are available from the article and its **Supplementary Materials**. The raw data of this article are not freely available due to ethical restrictions, but the scientific data (which don't include individual data) supporting the findings of this study are available up request.

## Ethics statement

The studies involving human participants were reviewed and approved by The Ethics Committee of the 2nd Affiliated Hospital of Dalian Medical University (2021-No.032). The patients/participants provided their written informed consent to participate in this study.

## Author contributions

GX, PG, RL and HZ conceived the initial concept and designed the study. XL, LY and PWG provided data collection. HK, WQ, PD and QL performed sample pretreatment and data preprocessing. XL, CX, PWG, QC, YL, YJ, ZZ, JY, XW and ZX carried out the metabolomic analysis, clinical laboratory test and sample collection. XL, PWG, JY, QW and JZ performed bioinformatic analysis. XL wrote the manuscript. GX and PG revised the paper. All authors contributed to the article and approved the submitted version.
