# Supplementary material for: Metabolomics acts as a powerful tool for comprehensively evaluating vaccines approved under emergency: a CoronaVac retrospective study
Source: Front Immunol. 2023 Jul 14;14:1168308. doi: 10.3389/fimmu.2023.1168308 (PMC10375237; doi:10.3389/fimmu.2023.1168308)
Supplement: Supplementary file 1 [file DataSheet_1.pdf]

Supplementary materials

Metabolomics acts as a powerful tool for comprehensively evaluating vaccines approved under emergency: a CoronaVac retrospective study

Xinyu Liu[1,2#], Congshu Xiao[3#], Pengwei Guan[1,2,4#], Qianqian Chen[1,2#], Lei You[1,2,4], Hongwei Kong[5], Wangshu Qin[1,2], Peng Dou[1,2], Qi Li[1,2], Yanju Li[6], Ying Jiao[7], Zhiwei Zhong[3], Jun Yang[1,2,4], Xiaolin Wang[1,2], Qingqing Wang[1,2,4], Jinhui Zhao[1,2,4], Zhiliang Xu[5], Hong Zhang[8*], Rongkuan Li[3*], Peng Gao[9,10*], Guowang Xu[1,2,4*]

1. CAS Key Laboratory of Separation Science for Analytical Chemistry, Dalian Institute of Chemical Physics, Chinese Academy of Sciences, Dalian, China
2. Liaoning Province Key Laboratory of Metabolomics, Dalian, China
3. Department of Infection, The Second Hospital of Dalian Medical University, Dalian, China
4. University of Chinese Academy of Sciences, Beijing, China
5. Hangzhou Health-Bank Medical Laboratory Co., Ltd. Hangzhou, China
6. Clinical laboratory, Affiliated Dalian Hospital of Shengjing Hospital of Chinese Medical University, Dalian, China
7. Nursing Department, Anshan Infectious Disease Hospital, Anshan, China
8. Internal Department, Women and Children's Hospital of Anshan City, Anshan, China
9. Clinical laboratory, The Second Hospital of Dalian Medical University, Dalian, China
10. Shanghai Institute for Biomedical and Pharmaceutical Technologies, Shanghai, China

# these authors contributed equally to this paper

*Corresponding authors: Guowang Xu, e-mail: xugw@dicp.ac.cn.
Tel./Fax: +86-411-84379530 (Lead contact)
Peng Gao, peng_med@yeah.net
Rongkuang Li, dalianlrk@126.com
Hong Zhang, aszh529@163.com

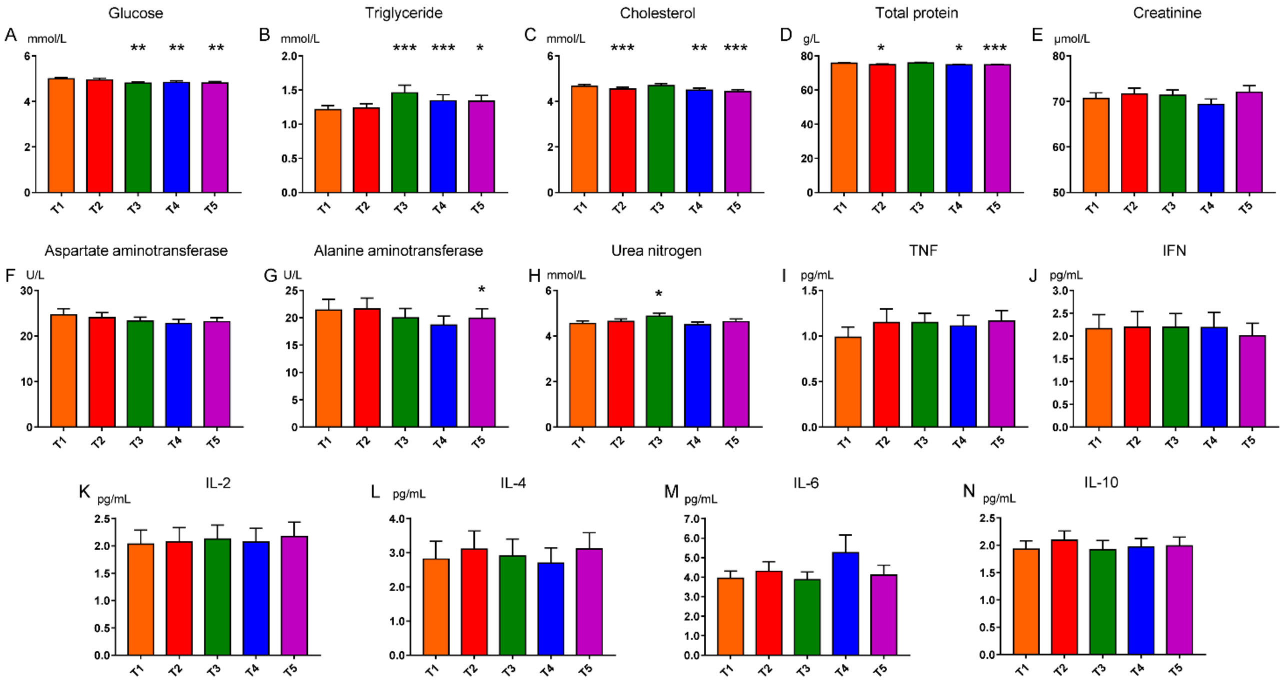

Fig. S1. Glucose, triglyceride, total protein, liver function and cytokine variations in response to vaccination in the discovery set. Paired nonparametric test, *: pFDR<0·05, **: pFDR<0·01, ***: pFDR<0·001, compared with T1. Typical 105 (21 subjects at five different time points) samples of discovery set were randomly selected and subjected to serum cytokine (TNF-α, IFN-γ, IL-2, IL-4, IL- 6, and IL-10) analysis ((T1 to T5: before the first vaccination (T1), 3 days after the first vaccination (T2), 3 days (T3), 15 days (T4), and 30 days (T5) after the second vaccination).

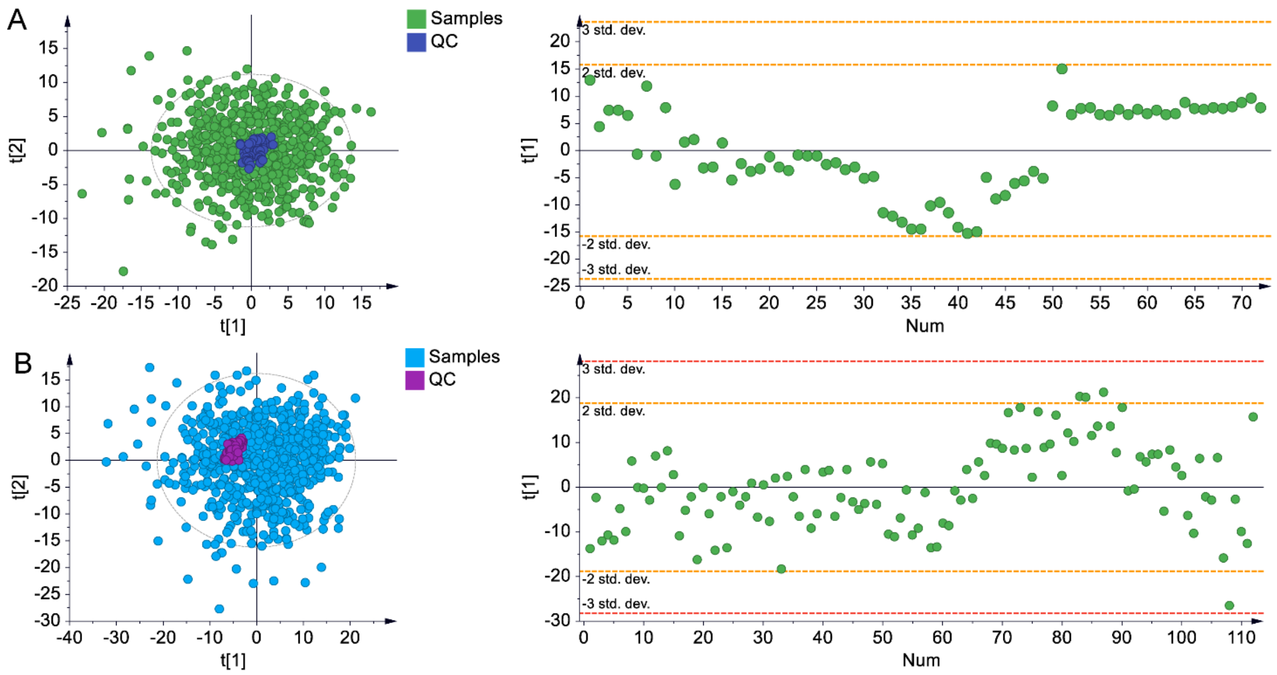

Fig. S2. Stability evaluation of metabolomics data (A) and lipidomics data (B).

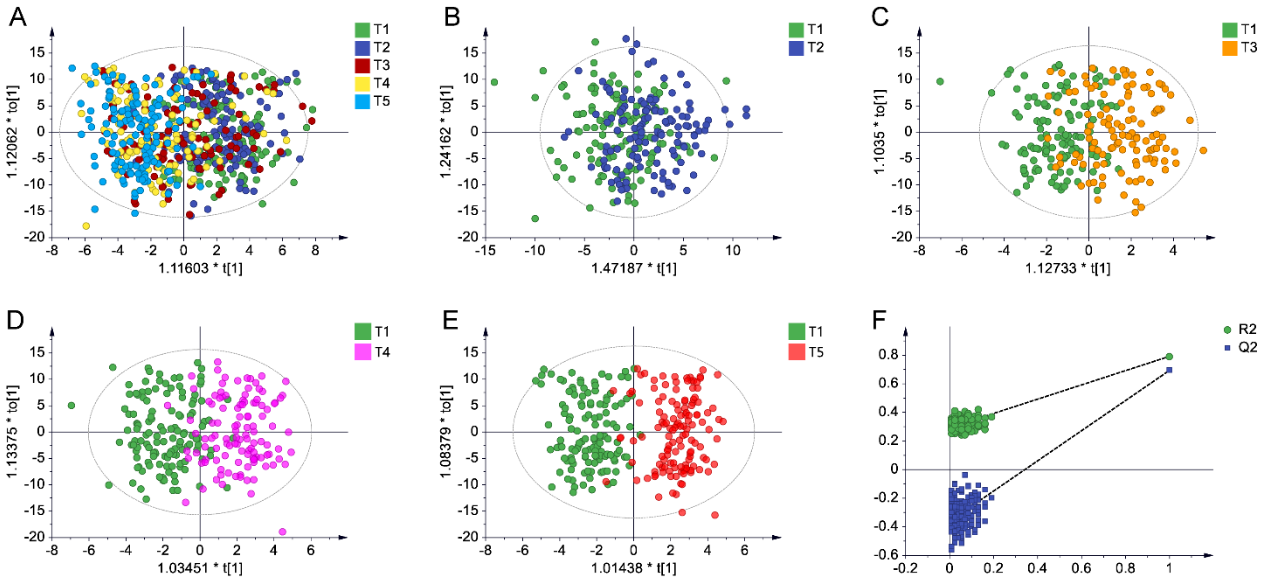

Fig. S3. Global metabolic difference of subjects with different metabolic variations in response to vaccination in validation set. A, Score plot of OPLS-DA model for all subjects at different sampling times (T1~T5). B-D, Score plot of OPLS-DA model for T1 vs. T2, T1 vs. T3, and T1 vs. T4. E-F, Score plot and cross validation of OPLS-DA model for T1 vs. T5. In A~E, UV scaling was performed, no overfitting was found.

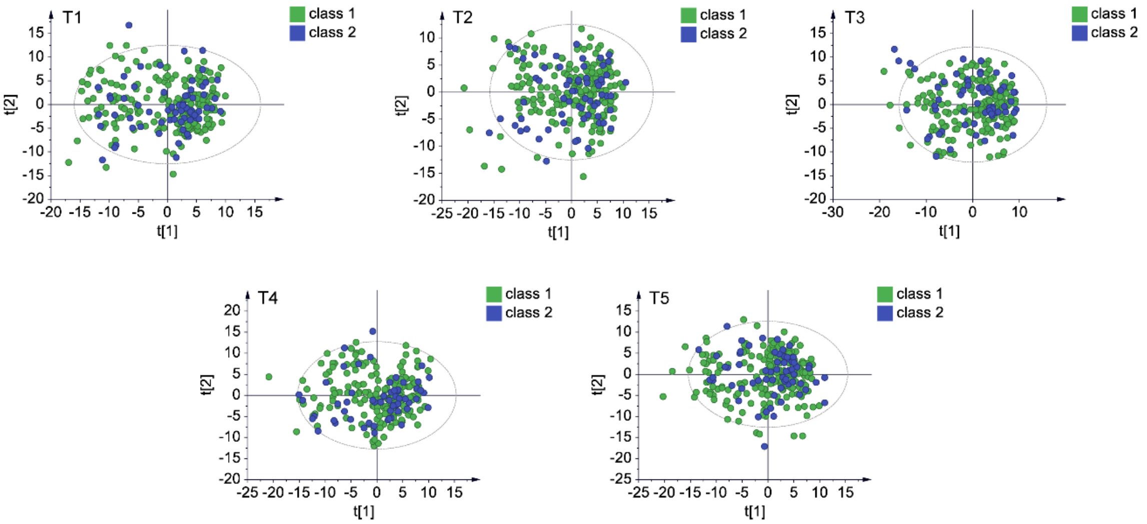

Fig. S4. PCA of the young (class 1, age <44) and old (class 2, age≥44) people metabolic profiling data from the 5 time points individually. Clearly, for any time point data, the young people and old people could not be separated in the score plot. T: time point.

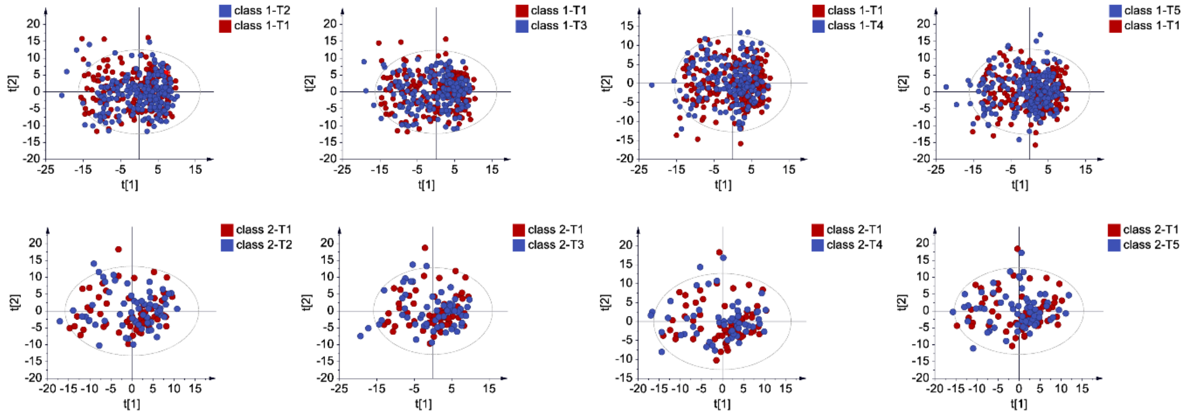

Fig. S5. Metabolic profiling data PCA of the young (class 1, age <44) and old (class 2, age≥44) people before (T1) and after (T2-T5) inoculation. Clearly, for any time point data, there was no separation between the data of before inoculation and after inoculation. T: time point.

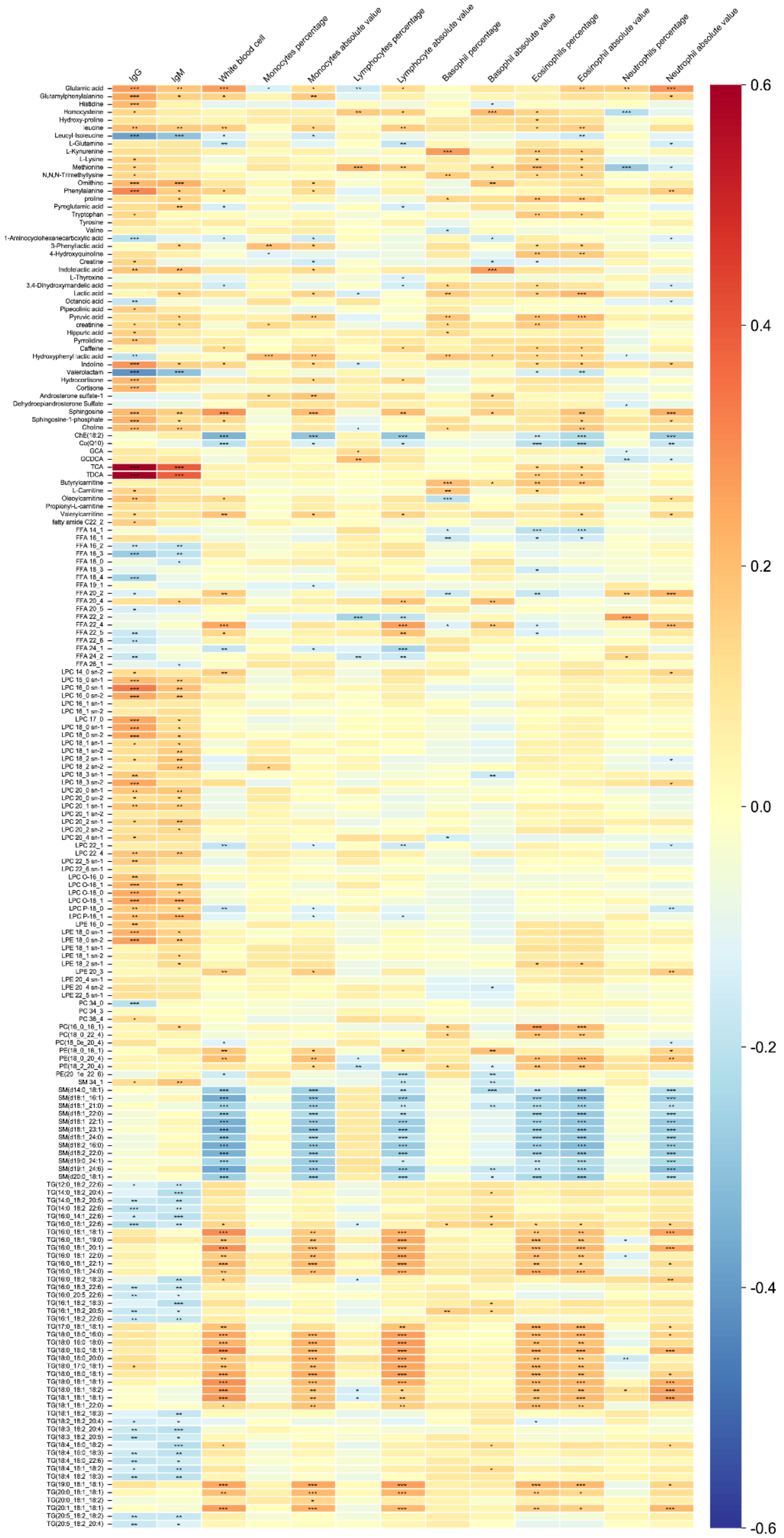

Fig. S6. Correlation between differential metabolites response to vaccination and immune related clinical parameters in the discovery set of T1 and T5 data. Spearman correlation coefficient, *: p<0·05, **: p<0·01, ***: p<0·001.

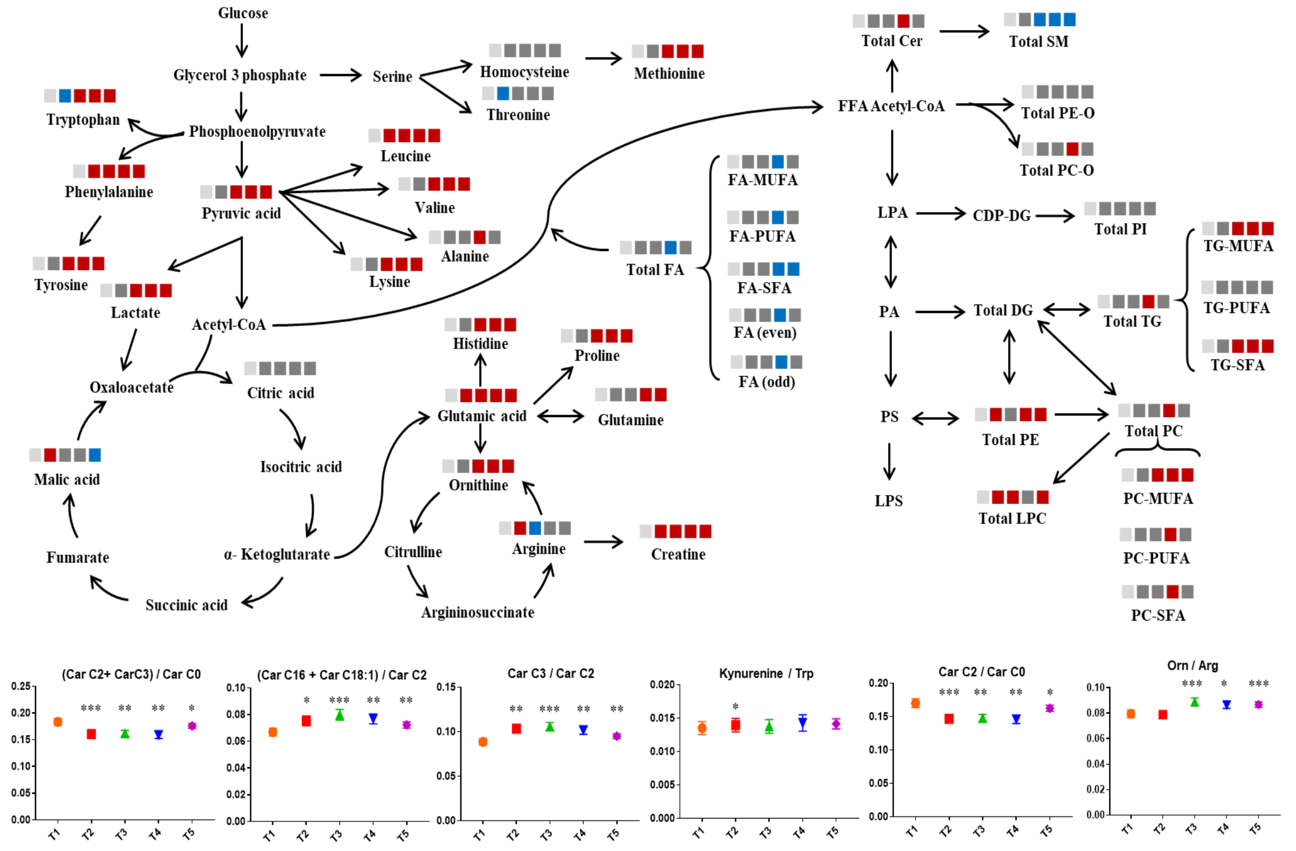

Fig. S7. Global metabolic pathway variations in response to vaccination based on differential metabolites between T1 and T5 in the discovery set. Paired nonparametric test, *: pFDR<0·05, **: pFDR<0·01, ***: pFDR<0·001, compared with T1. Pyruvic acid and lactic acid were significantly elevated after 3 days of the second vaccination, while tricarboxylic acid cycle concurrently down-regulated after vaccination. Decreased ratio of acetylcarnitine (Car C2:0) and/or propionylcarnitine (Car C3:0) to carnitine implied that β-oxidation rate reduced after vaccination. Ratio of (Car C16:0+Car C18:1) to Car C2:0 was associated with mitochondrial long-chain fatty acid oxidation, such changes also provided evidence to metabolic shift from energy supply to building blocks providing. Amino acid metabolism, including aromatic amino acid metabolism, branched chain amino acid metabolism, glutamate metabolism, etc., was significantly up-regulated. Lipid species were represented by the sum of individual lipids in each specific class.

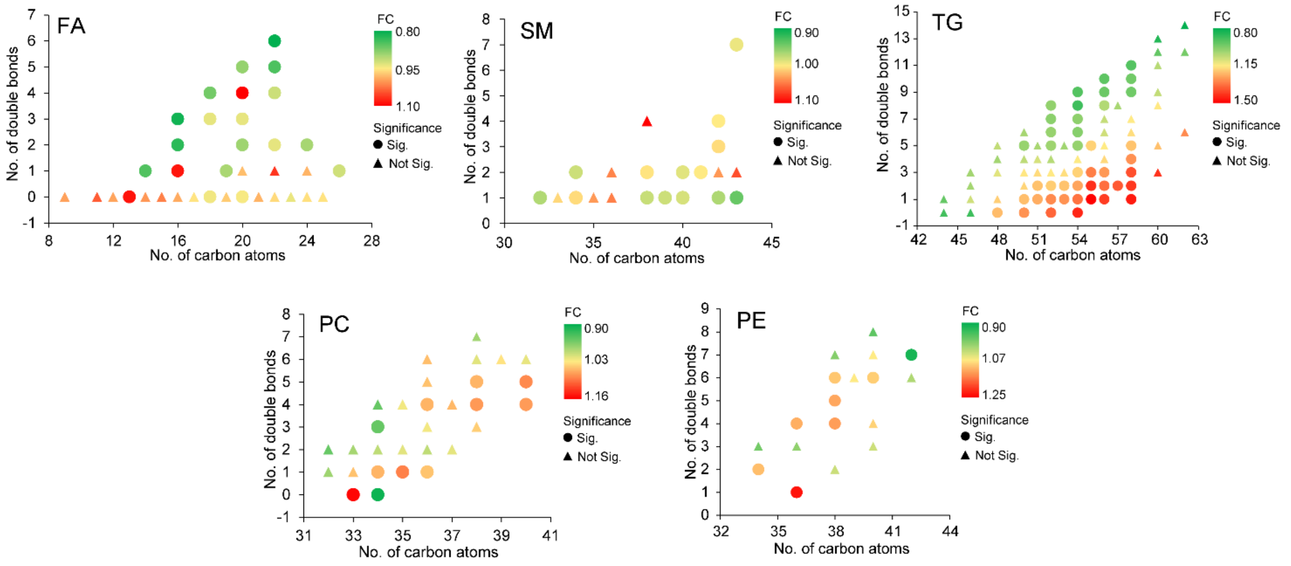

Fig. S8. Alteration of differential lipids by carbon number and double bond number in discovery set (FC=T5/T1, nonparametric test, p<0.05 was used).

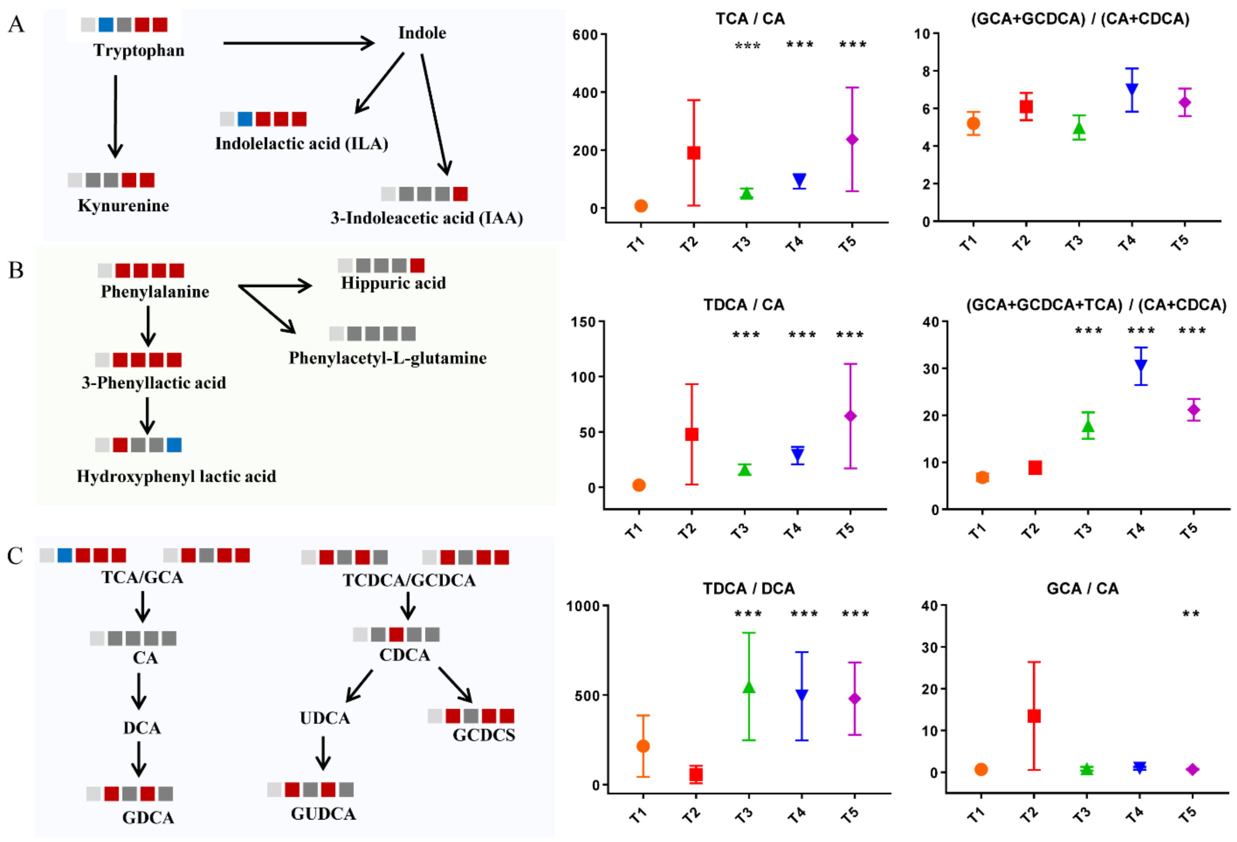

Fig. S9. Intestinal flora metabolism related pathway variations in response to vaccination based on differential metabolites between T1 and T5 in the discovery set. Paired nonparametric test, *: pFDR<0·05, **: pFDR<0·01, ***: pFDR<0·001, compared with T1. Ratio of taurine/glycine-conjugated bile acids to unconjugated bile acids indicated enzymatic activity of bile acid CoA ligase and bile acid CoA: amino acid N-acyltransferase activity in the liver. Additionally, primary bile acid (eg. cholic acid) could be converted to secondary bile acid (eg. deoxycholic acid) and conjugated secondary bile acid (eg. glycodeoxycholic acid) by gut bacteria. Furthermore, intestinal microbiota could regulate the activity of related enzymes in bile acid metabolic pathway. Tryptophan and phenylalanine metabolism were believed to be associated with gut microbes, tryptophan and its metabolites (kynurenine, indolelactic acid (ILA), 3-indoleacetic acid (IAA)) substantially raised after vaccination. Similarly, phenylalanine and its metabolites (3-phenyllactic acid and hippuric acid) also memorably increased after vaccination.

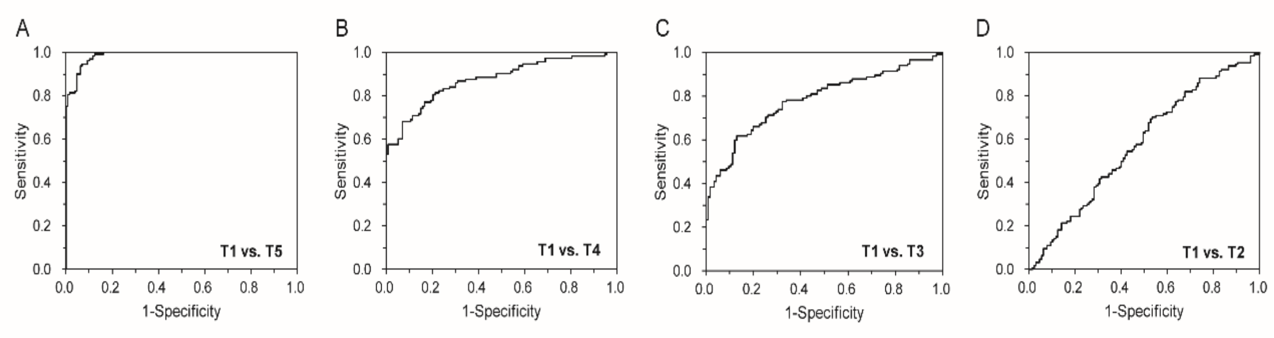

Fig. S10. ROC curves of the metabolic markers for the evaluation of immune response between T5, T4, T3, or T2 and T1 in the validation set.

Table S1. Serum antibody production in response to vaccination in the discovery and validation sets, respectively.

Time point	Antibody (IgG, IgM)									
T2: 3 Days After 1st Injection	+,+		+,-		-,+		-,-		Total subjects	
	Discovery	Validation	Discovery	Validation	Discovery	Validation	Discovery	Validation	Discovery	Validation
Number	3	3	3	6	30	33	128	121	164	163
%	1.83%	1.84%	1.83%	3.68%	18.29%	20.25%	78.05%	74.23%	327	
T3: 3 Days After 2nd Injection	+,+		+,-		-,+		-,-		Total subjects	
	Discovery	Validation	Discovery	Validation	Discovery	Validation	Discovery	Validation	Discovery	Validation
Number	3	11	15	12	42	36	102	105	162	164
%	1.85%	6.71%	9.26%	7.32%	25.93%	21.95%	62.96%	64.02%	326	
T4: 15Days After 2nd Injection	+,+		+,-		-,+		-,-		Total subjects	
	Discovery	Validation	Discovery	Validation	Discovery	Validation	Discovery	Validation	Discovery	Validation
Number	65	66	38	57	11	6	31	21	145	150
%	44.83%	44.00 %	26.21%	38.00%	7.59%	4.00%	21.38%	14.00%	295	
T5: 1 Month After 2nd Injection	+,+		+,-		-,+		-,-		Total subjects	
	Discovery	Validation	Discovery	Validation	Discovery	Validation	Discovery	Validation	Discovery	Validation
Number	62	62	57	64	18	7	27	33	164	166
%	37.80%	37.35%	34.76%	38.55%	10.98%	4.22%	16.46%	19.88%	330	

Data of very few sampling points of several samples were lost, hence, not included in this calculation.

Table S2. Clinical parameters variations in response to vaccination in the discovery set (subjects with antibody positive status at T5 were involved in calculation).

Clinical Parameters	Time Point 1 (T1)	Time Point 2 (T2)	Time Point 3 (T3)	Time Point 4 (T4)	Time Point 5 (T5)	Reference Range (Female; Male)
Female/Male (74/60)						
Age (34.29±14.91)						
BMI (23.22±3.67)						
Red blood cell	4.76±0.48	4.77±0.45	4.73±0.44	4.71±0.45	4.7±0.51***	3·8~5·1 10^12/L; 4·3~5·8 10^12/L
Hemoglobin	143.87±15.46	144.31±15.62	142.06±16.23*	140.32±14.24***	143.45±16.66*	115~150g/L; 130~175g/L
Platelets	259.15±60.92	255.57±58.4*	259.59±59.53	265.93±59.78***	257.27±59.72	125~350 10^9/L
Large platelet volume	0.26±0.06	0.25±0.06**	0.27±0.07**	0.29±0.06***	0.26±0.06	0·13~0·43 %
Percentage of monocytes	7.2±1.73	9.47±2.43***	7.96±1.89***	7.57±1.73*	7.34±1.82	3~10 %
Erythrocyte distribution width CV	12.96±1.22	13.02±1.23*	12.74±1.39**	12.67±1.14***	13.15±1.39***	10·9~15·4 %
Erythrocyte distribution width SD	41.43±3.13	41.61±3.09	40.97±2.87*	40.7±2.85***	42.03±3.16**	39~46 fL
Hematocrit	0.43±0.04	0.43±0.04	0.42±0.04*	0.42±0.04***	0.42±0.04***	0·35~0·45 L/L; 0·4~0·5 L/L
Percentage of lymphocytes	35.4±6.98	39.18±8.25***	36.72±6.75	36.42±7.67	35.83±7.48	20~50 %
Mean Hb content of erythrocytes	30.27±2.34	30.29±2.28	30.05±2.33*	29.83±1.81***	30.57±2.26***	27~34 pg
Mean erythrocyte Hb concentration	335.69±10.54	335.59±10.28	336.18±12.37	337.33±8.62*	337.93±9.84***	316~354 g/L
Mean red blood cell volume	90.09±5.65	90.18±5.6	89.29±5.25**	88.4±4.59***	90.37±5.4	82~100 fL
Mean platelet volume	9.97±1.18	9.89±1.23	10.46±1.74***	10.81±1.07***	9.98±1.1	9·4~12·5 fL
Basophil percentage	0.64±0.32	0.59±0.28	0.67±0.33	0.64±0.32	0.67±0.44	0~1 %
Percentage of eosinophils	2.14±1.75	2.12±1.46	2.5±1.85**	2.18±1.5*	2.39±1.95*	0·4~8 %
Platelet volume width	15.18±2.47	15.26±2.41	13.7±2.91***	13.12±2.55***	15.19±2.41	15·5~17·1 fL
Percentage of nucleated red blood cells	0.11±0.14	0.09±0.09	0.06±0.08*	0.03±0.07***	0.25±0.36***	——/100WBC
Absolute value of nucleated red blood cells	0.007±0.008	0.006±0.005	0.003±0.004**	0.002±0.004***	0.015±0.019***	——/10^9/L
Percentage of neutrophils	54.62±7.6	48.64±9.1***	52.15±7.71**	53.18±8.29*	53.77±8.11	40~75 %
Albumin	50.3±3.21	48.99±2.61***	46.99±2.53***	46.42±2.78***	45.3±2.17***	40~55 g/L
Total bilirubin	10.94±4.28	10.22±4.14	10.64±4.89	10.88±4.22	11.05±4.52	0~26 μmol/L

*: pFDR<0·05, **: pFDR<0·01, ***: pFDR<0·001, compared with T1. Very few sampling points of several samples were lost, hence, not included in the subsequent analysis. The statistics of Female/Male ratio, age and BMI were based on the data at T1 *vs.* T2 time point.

Table S3. Performance of serum metabolite panel in the discovery set and validation set.

Data Set and comparison	Items	T1 *vs.* T5	T1 *vs.* T4	T1 *vs.* T3	T1 *vs.* T2
Discovery set	AUC	0·960	0·833	0·775	0·577
	Sensitivity	89·2%	80·4%	82·6%	-
	Specificity	87·7%	73·2%	59·5%	-
	Subjects (n)	130	112	121	134
	Age	33.53±14.28	34.38±14.92	34.87±15.37	34.29±14.91
	BMI	23.16±3.66	22.82±3.48	23.2±3.68	23.22±3.67
Validation set	AUC	0·986	0·874	0·783	0·575
	Sensitivity	93·8%	70·8%	79·1%	-
	Specificity	93·0%	86·7%	59·1%	-
	Subjects	130	113	116	128
	Age	33.79±13.87	34.12±13.72	33.6±13.83	33.72±13.87
	BMI	23.29±4.1	23.18±4.01	23.16±4.14	23.27±4.1
